# Altered complexity of resting-state BOLD activity in Alzheimer’s disease-related neurodegeneration: a multiscale entropy analysis

**DOI:** 10.18632/aging.103463

**Published:** 2020-07-10

**Authors:** Ping Ren, Manxiu Ma, Guohua Xie, Zhiwei Wu, Donghui Wu

**Affiliations:** 1 Shenzhen Mental Health Center, Shenzhen, Guangdong, China; 2 Shenzhen Kangning Hospital, Shenzhen, Guangdong, China; 3 Center for Neurobiology Research, Fralin Biomedical Research Institute at VTC, Virginia Tech, Roanoke, VA 24016, USA

**Keywords:** complexity, Alzheimer’s disease, resting-sate fMRI, multiscale entropy, amnestic mild cognitive impairment

## Abstract

Brain complexity, which reflects the ability of the brain to adapt to a changing environment, has been found to be significantly changed with age. However, there is less evidence on the alterations of brain complexity in neurodegenerative disorders such as Alzheimer’s disease (AD). Here we investigated the altered complexity of resting-state blood oxygen level-dependent signals in AD-related neurodegeneration using multiscale entropy (MSE) analysis. All participants were recruited from the Alzheimer’s Disease Neuroimaging Initiative, including healthy controls (HC, n=62), amnestic mild cognitive impairment (aMCI, n =81) patients, and Alzheimer’s disease (AD, n=25) patients. Our results showed time scale-dependent MSE differences across the three groups. In scale=1, significantly changed MSE patterns (HC>aMCI>AD) were found in four brain regions, including the hippocampus, middle frontal gyrus, intraparietal lobe, and superior frontal gyrus. In scale=4, reversed MSE patterns (HC<aMCI<AD) were found in the middle frontal gyrus and middle occipital gyrus. Furthermore, the values of regional entropy were significantly associated with cognitive functions positively on the short time scale, while negatively on the longer time scale. Our findings suggest that MSE could be a reliable measure for characterizing brain deterioration in AD, and may provide insights into the neural mechanism of AD-related neurodegeneration.

## INTRODUCTION

The human brain is a nonlinear dynamic system, which needs to keep flexible or complex to adapt to the external changing environment. Neuroimaging studies have demonstrated that the variability of brain signals is more than intrinsic spontaneous noise, and is closely linked to different cognitive states [1, 2]. Thus the loss of complexity in brain activity may lead to neural inefficiency and cognitive deficits, which substantially increase the risk of the susceptibility for many mental disorders. Previous study has shown that older adults exhibited significantly decreased complexity of resting brain activity compared with young adults [3], suggesting declined brain functions and impaired adaptability to environment with age. Alzheimer’s disease (AD) is the most common form of dementia in aging most often characterized by cognitive deficits in multiple domains, including episodic memory, executive function, and decision-making [4, 5]. Although substantial neuroimaging studies have reported brain alterations in AD using various measures, there has been less study on how the complexity of brain activity changes in AD-related neurodegeneration.

To quantify the complexity in a dynamic system, entropy is developed to measure the nonlinear characteristics of time series data, which has been successfully applied in scientific and clinical researches [6, 7]. In several AD-related neuroimaging studies, converging evidence has shown the abnormality of altered complexity in brain activity, associated with impaired cognitive and psychosocial functions. For example, electroencephalography (EEG) studies have reported approximate entropy significantly decreased in AD patients, suggesting a reduced irregularity in brain activity [8, 9]. As a modification of approximate entropy, sample entropy is less sensitive to the change of data length, and has been found significantly decreased in AD patients as well [10]. Likewise, entropy analysis in magnetoencephalography (MEG) studies reported significantly decreased complexity or increased regularity of brain activity in AD [11, 12]. Different from using a single temporal scale in approximate entropy and sample entropy analysis, multiscale entropy (MSE) has been developed to assess the irregularity of a time-series signal in multiple time scales, providing a more informative measurement in exploring age-related brain deterioration [3, 13].

In recent years, increasing numbers of studies have been focusing on the dynamic analysis of blood-oxygen-level-dependent (BOLD) signals derived from resting-state fMRI (rs-fMRI) data. Compared with EEG and MEG, fMRI provides high spatial resolution in understanding regional activity and interregional connections. Using resting-state fMRI, accumulating evidence has revealed AD-related dysfunctions in multiple brain regions, including the prefrontal cortex (PFC), hippocampus, parietal lobe, and temporal lobe [14, 15]. Functional connectivity analysis indicates that decreased global connections and increased local connections were found in amnestic mild cognitive impairment (aMCI) and AD [16, 17]. In addition to the conventional fMRI analyses, recent studies have reported entropy of spontaneous BOLD signals could be a sensitive biomarker for assessing the complexity and synchronicity of brain activity in normal aging [18] and psychiatric disorder [19]. Taken together, the disrupted regional brain activities and network connectivities implicate the abnormal dynamics of spontaneous brain activity in AD, suggesting an altered BOLD complexity may be closely linked to the etiology of AD.

In the current study, we investigated the changes in fMRI signal complexity in aMCI and AD patients compared with healthy controls (HC) by examining MSE of resting-state BOLD signals. We hypothesized that MSE would be altered in multiple brain regions in aMCI and AD groups, and was associated with AD-related cognitive decline. Moreover, we speculated that the changes of MSE patterns in AD would depend on different time scales.

## RESULTS

### Defining parameters in MSE analysis

**Figure 1:**
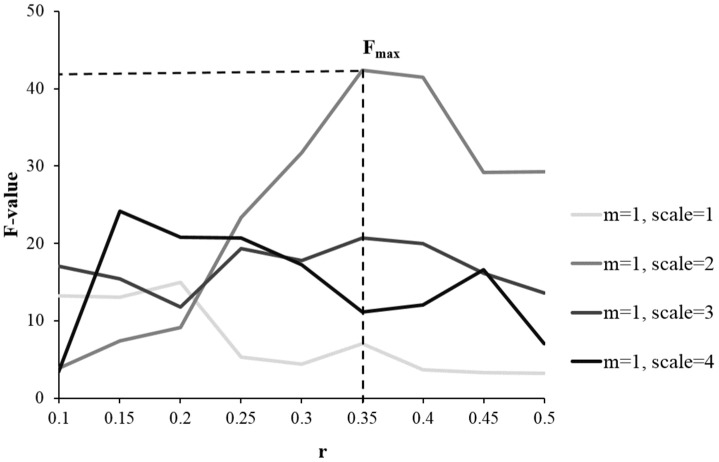
**Examining the sensitivity of MSE parameters in differentiating the HC, aMCI, and AD groups using one-way ANOVA.** Compared with other parameters, the combination of *m* = 1, *r* = 0.35, and scale = 2 gave the largest total F scores, showing the highest discrimination power of detecting the differences across the three groups.

**Figure 2:**
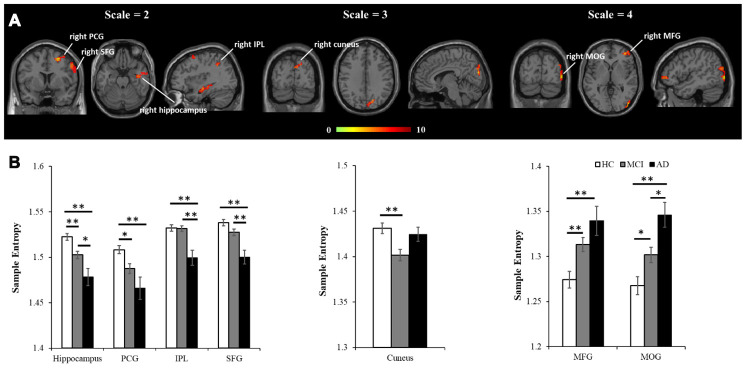
**MSE differences across the three groups using voxel-wise ANCOVA, controlled for age, education, head motion, and GM.** (**A**) Significant differences of MSE were observed in multiple brain regions, including four regions in scale = 2 (the right hippocampus, right PCG, right IPL and right SFG), one region in scale = 3 (the right cuneus), and two regions in scale = 4 (the MFG and MOG). All the statistical results were corrected for multiple comparisons (p < 0.05, AlphSim correction). (**B**) The peak entropy value within each region was extracted and used to examine the differences between each pair of groups (p<0.05, FDR correction). Note: PCG, precentral gyrus; IPL, intraparietal lobe; SFG, superior frontal gyrus; MFG, middle frontal gyrus; MOG, middle occipital gyrus; GM, gray matter. *, p<.05; **, p<.01.

Based on analyzing 90 regions in AAL template, results from one-way ANOVA showed MSE parameters of *m* = 1, *r* = 0.35 and scale = 2 with the largest total F scores (F = 42.4), giving the highest sensitivity in differentiating the HC, aMCI and AD groups (see Figure 1). Then these parameters were applied to calculate the individual’s entropy map for the subsequent whole-brain voxel-wise analysis. A voxel-wise ANCOVA was used to examine the differences across the three groups, and showed significant MSE changes in four brain regions (p < 0.05, AlphaSim correction), including the right hippocampus, right precentral gyrus (PCG), right intraparietal lobe (IPL), and right superior frontal gyrus (SFG) (see Figure 2A left and Table 2). Post-hoc comparisons were performed to examine the differences between each paired group. In the hippocampus and PCG, both aMCI and AD groups showed significantly decreased entropy than the HC group (p < 0.05, FDR correction). In the IPL and SFG, the AD group showed significantly decreased entropy compared with HC and MCI groups (p < 0.05, FDR correction, see Figure 2B left).

**Table 1:** Demographics and characteristics of participants.

	**HC (n = 62)**	**aMCI (n = 81)**	**AD (n = 25)**	**p value**
**Age**	74 ± 6	74 ± 7	74 ± 6	0.79
**Male (n)**	28	43	13	0.63
**Education (Years)**	16 ± 3	16 ± 3	15 ± 3	0.42
**MOCA**	26 ± 2	23 ± 3 *	16 ± 5 *,^#^	< 0.01
**MEM**	1.0 ± 0.6	0.3 ± 0.7 *	-0.8 ± 0.7 *,^#^	< 0.01
**EF**	0.8 ± 0.7	0.3 ± 0.9 *	-0.9 ± 0.9 *,^#^	< 0.01
**Head motion**	0.18 ± 0.11	0.19 ± 0.16	0.18 ± 0.13	0.96

Data are presented as means ± standard deviations for the HC, aMCI, and AD groups. *, p<0.05 compared with HC; #, p<0.05, compared with MCI. Note: HC, healthy control; aMCI, amnestic mild cognitive impairment; AD, Alzheimer’s disease; MOCA, Montreal Cognitive Assessment; MEM, memory; EF, executive function.

**Table 2:** Brain regions with significant MSE differences across HC, aMCI, and AD.

**Region**	**Peak value**	**Cluster (voxels)**	**MNI coordinates**	**p value**
**Time Scale=2**				
Right hippocampus	14.5	262	39, -15, -15	<0.001
Right PCG	8.7	71	57, 6, 42	0.002
Right IPL	10.7	59	30, -48, 45	0.014
Right SFG	13.5	54	24, 6, 54	0.028
**Time Scale=3**				
Right cuneus	7.2	52	6, -84, 21	0.04
**Time Scale=4**				
Right MFG	8.5	53	39, 48, 0	0.04
Right MOG	10.1	106	48, -84, -3	<.001

Significant MSE changes across groups by using ANCOVA, controlled for age, education, head motion, and GM (p < 0.05, AlphaSim correction). Note: MNI, Montreal Neurological Institute; PCG, precentral gyrus; IPL, intraparietal lobe; SFG, superior frontal gyrus; MFG, middle frontal gyrus; MOG, middle occipital gyrus; GM, gray matter.

### MSE analysis in different time scales

Further analyses examined how the altered MSE patterns changed with time scales. In scale = 1, there was no significant difference across the HC, aMCI, and AD groups. In scale = 3, entropy in the right cuneus showed significant difference across the three groups (see Figure 2A middle, Table 2). A post-hoc analysis showed that the aMCI group has significantly decreased entropy than the HC group (p < 0.05, FDR correction). In scale = 4, entropy was found significantly different in two brain regions, including the right middle frontal gyrus (MFG) and right middle occipital lobe (MOG) (see Figure 2A right, Table 2). A post-hoc analysis showed AD > HC and aMCI > HC in the right MFG, and AD > aMCI > HC in the right MOG (p < 0.05, FDR correction).

**Figure 3:**
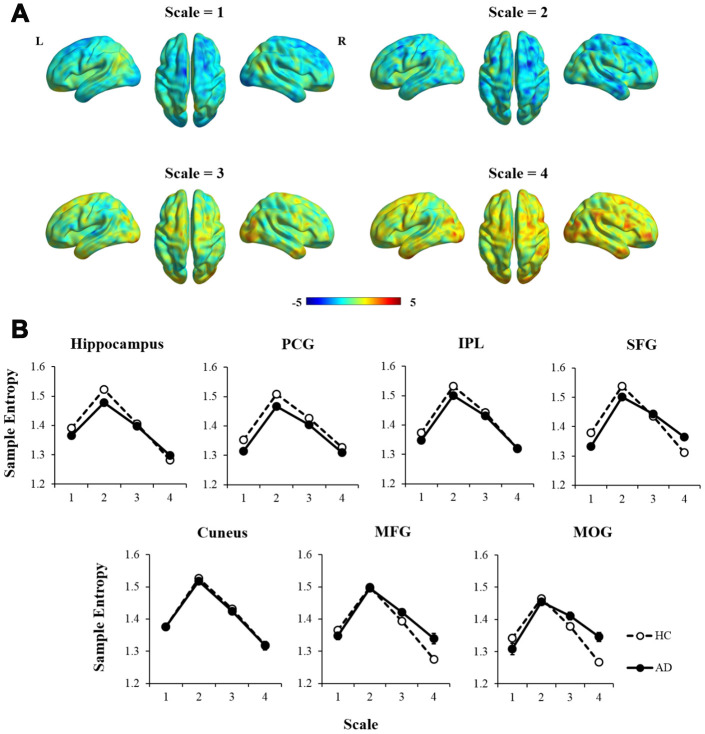
**The comparison of MSE patterns between AD and HC in different time scales using independent two-sample t-test, controlled for age, education, head motion, and GM.** (**A**) In scale = 1 and 2, most of the brain regions showed decreased entropy in AD compared with HC. In scale = 3 and 4, the statistical maps tended to show reversed patterns of MSE. (**B**) MSE of each region was extracted and showed significant interaction effects of group × scale in the hippocampus, SFG, MFG, and MOG (p < 0.01). Note: PCG, precentral gyrus; IPL, intraparietal lobe; SFG, superior frontal gyrus; MFG, middle frontal gyrus; MOG, middle occipital gyrus; GM, gray matter.

Additionally, an independent two-sample t-test was performed to examine how the MSE patterns of AD versus HC changed with time scales (see Figure 3A). To better represent the trajectory of entropy changes, we didn’t apply any criterion to the statistic maps. Compared with HCs, AD patients showed widespread regions with decreased entropy in short time scales (scale =1 and 2), followed by more regions with increased entropy in longer time scales (scale=3 and 4). The entropy values within the brain regions defined in the ANCOVA were extracted to show the time scale-dependent MSE changes in the HC and AD groups, respectively (see Figure 3B). After controlling for age, education, GM, and head motion, a repeated measures analysis showed significant interaction effects of group × scale in the hippocampus, SFG, MFG, and MOG (uncorrected p < 0.01).

### Correlation analysis for MSE

**Figure 4:**
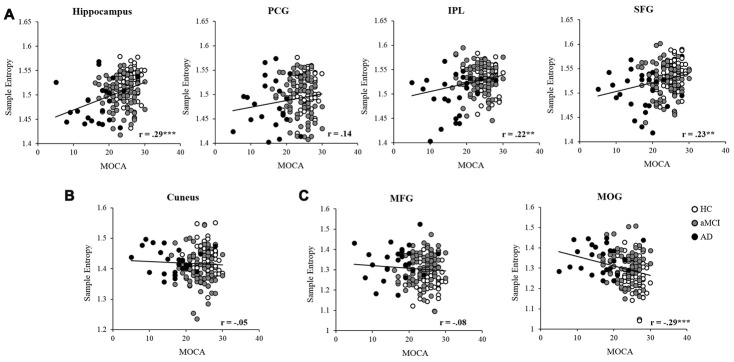
**Partial correlation was applied to examine the relationships between MSE and MOCA in the entire sample, controlled for age, education, head motion, and GM.** The entropy showed significant positive correlations in the hippocampus, IPL, and SFG (**A**), and negative correlation with the MOG (**C**). While no significant correlation was found between MOCA and entropy in the PCG (**A**), cuneus (**B**), and MFG (**C**). Note: PCG, precentral gyrus; IPL, intraparietal lobe; SFG, superior frontal gyrus; MFG, middle frontal gyrus; MOG, middle occipital gyrus; GM, gray matter. *, p<.05; **, p<.01; ***, p<.001.

Partial correlation was applied to examine the relationships between MSE of BOLD signals and neuropsychological assessments in the entire sample, controlled for age, education, GM, and head motion (see Table 3). In scale = 2, the results showed that MOCA was positively correlated with MSE in the hippocampus, IPL, and SFG (see Figure 4A). The MEM and EF were positively correlated with MSE in the hippocampus, IPL and SFG separately. Also, the EF was positively associated with MSE in the MFG as well. In scale = 3, there was no significant correlation found in the cuneus (see Figure 4B). In scale = 4, negative correlations were found between MOCA and MSE in the MOG (see Figure 4C). In addition, the MEM and EF were negatively correlated with MSE in the MFG and MOG separately

**Table 3:** Relationships of MSE with neuropsychological assessments.

**Region**	**MOCA**	**MEM**	**EF**
**Time scale = 2**			
Right hippocampus	.29 ***	.27 ***	.19**
Right MFG	.14	.16 *	.23 **
Right IPL	.22 **	.18 **	.18 *
Right SFG	.23 **	.26 ***	.27 ***
**Time scale = 3**			
Right cuneus	-.05	.07	.09
**Time scale = 4**			
Right MFG	-.08	-.20 **	-.18 *
Right MOG	-.29 ***	-.20 **	-.26 ***

Partial correlation showed the relationships between entropy and MOCA, MEM, EF in the entire sample, controlled for age and education. Data represents the correlation coefficient (r). Note: MNI, Montreal Neurological Institute; PCG, precentral gyrus; IPL, intraparietal lobe; SFG, superior frontal gyrus; MFG, middle frontal gyrus; MOG, middle occipital gyrus. *, p<0.05; **, p<0.01; ***, p<0.001.

## DISCUSSION

In the current study, we investigated the altered complexity of resting-state BOLD signals in AD-associated neurodegeneration, and its relationships with declined cognitive functions. Compared with HCs, AD patients showed significantly decreased MSE of BOLD signals for scale = 1, while increased MSE for scale = 4. Likewise, aMCI group showed moderately decreased MSE on a short scale, and increased MSE on a longer scale. Interestingly, we found unexpected non-monotonic MSE changes (HC > aMCI <AD) in the cuneus in scale = 3, suggesting a complicated pattern of brain functional alterations in AD progression. Additionally, the correlation analyses showed that the relationships between BOLD complexity and cognitive functions depended on different time scales. Our study found that MSE of BOLD activity successfully characterized the brain deterioration of AD-related neurodegeneration, which may be a sensitive biomarker for AD diagnosis and intervention.

The abnormal BOLD complexity of multiple brain regions in the patient groups may indicate disrupted brain connections and declined cognitive functions. On a short time scale (scale = 2), aMCI and AD exhibited significantly changed MSE values within four brain regions, including the SFG, PCG, hippocampus, and IPL. Converging evidence has shown that AD-related neurodegenerative disorders show significantly functional and structural disruptions of the hippocampus, which lead to typical memory deficits [20, 21]. Since entropy in short time scales has been found associated with local information processing [22], the reduced entropy may be due to disrupted regional activity or local connectivity of the hippocampus in AD [23, 24]. As a core hub of the default mode network (DMN), the IPL showed decreased low-frequency oscillation in AD, suggesting the hypoactivity of the DMN [25]. In addition, reduced regional activity and disrupted connections of the SFG and PCG have been found in individuals at risk for AD [16, 26]. In line with these studies, here the decreased pattern of BOLD complexity on a short time scale confirmed that these brain regions were vulnerable to AD pathology, especially for the hippocampus and PCG. In contrast to the findings on the short scale, several brain regions, such as the MFG and MOG, exhibited significantly increased entropy in the aMCI and AD groups on the longer scale (scale = 4). Similarly, findings from EEG study also showed entropy was decreased on the short scales and increased on the longer scales in AD compared with HC [27]. As well, several neuroimaging studies have reported abnormal hyperactivity and enhanced connectivity in the frontal and occipital regions in AD brain, which may be due to a compensation mechanism [28, 29]. It is noteworthy that we found an unexpected non-monotonic pattern of MSE change (HC > aMCI < AD) in the cuneus (scale = 3). A similar phenomenon has been found in previous AD-related studies as well. For example, Dickerson et al. reported hyperactivity of the hippocampus in aMCI compared with HC and AD in the face-name encoding task [30]. Using graph theoretical analyses, Seo et al. found that functional association between neighboring brain regions was most severely altered in aMCI stage and gradually re-increase in AD [31]. Taken together, our results indicated that time scale is an important factor in studying the biological mechanism of AD, and aMCI may be a more complicated state rather than a simple transitional stage between normal aging and dementia.

To further assess the changes of BOLD complexity over cognitive functions, correlation analyses were applied to examine the relationships between BOLD complexity and neuropsychological assessments. We found time scale-dependent correlations between altered entropy and cognitive deficits. On a short time scale, the entropy in the hippocampus, IPL, and SFG were positively correlated with cognitive functions, showing decreased entropy with impaired adaptability to cognitive demands in the AD group. While on a longer scale, the entropy values of the MFG and MOG were negatively associated with cognitive functions, representing higher entropy associated with more cognitive deficits in AD. Therefore, we speculate the higher regional entropy in AD on longer scales may be due to increased uncorrelated randomness according to Yang’s previous study [19]. Notably, although the correlations between cognitive deficits and altered entropy were highly significant in some regions such as the right hippocampus and right SFG, we must be cautious to interpret the results due to the small correlation coefficients (r = 0.18 - 0.29). A more appropriate way to evaluate the effect size is to calculate squared correlations, which represent explained percentage of variance. Here the small range of effect size (r^2^ = 3.2% - 8.4%) implies the relationships between regional entropy and cognitive functions are quite limited.

Several limitations need to be considered in the current study. First, the MSE analysis was limited to the short length of BOLD time series (130 time points) and relative low temporal resolution (TR = 3000 ms). Therefore, it would be difficult to compare our results with previous findings from EEG-based MSE analysis. Although previous studies have shown that sample entropy and MSE were successfully applied in short BOLD data [13, 32], longer time series data would be better to characterize the profiles of brain entropy. In recent years, a parallel imaging technique named multiband imaging has been widely used to acquire more data points with a short repetition time (TR < 1000 ms), which may partially solve this problem [33]. Second, we didn’t subdivide the aMCI group into early and late stages, which have been found changed differently in regional activities [14]. Future studies need to validate whether the patterns of altered MSE are different between the two stages. Third, the numbers of subjects were unbalanced across the HC, aMCI, and AD groups due to the limitation of ADNI dataset, thus it is worth replicating the current analysis in a well-balanced dataset in the future. Lastly, since the current study only examined the relationships between MSE changes and cognitive assessments, further studies need to involve more AD-related biochemical measurements, such as, but not limited to pro-inflammatory cytokines, as well as levels of hydrogen sulfide.

In summary, the altered MSE of resting-state BOLD activity may be linked to AD-related brain deterioration, reflecting the impaired capability of adapting to the external changing environment. Moreover, the altered BOLD complexity and its correlation with cognitive deficits are dependent on time scales. Our findings confirm the crucial role of the temporal dimension in understanding the neural substrates of AD, and suggest that MSE of brain activity may be a potential biomarker for AD diagnosis and intervention.

## MATERIALS AND METHODS

### ADNI dataset

Data used in the preparation of this research were obtained in April 2015 from the Alzheimer’s Disease Neuroimaging Initiative (ADNI) database (adni.loni.ucla.edu). The ADNI was launched in 2003 by the National Institute on Aging (NIA), the National Institute of Biomedical Imaging and Bioengineering (NIBIB), the Food and Drug Administration (FDA), private pharmaceutical companies and non-profit organizations, as a $60 million, 5- year public-private partnership. The primary goal of ADNI has been to test whether serial magnetic resonance imaging (MRI), positron emission tomography (PET), other biological markers, and clinical and neuropsychological assessment can be combined to measure the progression of MCI and AD. Determination of sensitive and specific markers of very early AD progression is intended to aid researchers and clinicians develop new treatments and monitor their effectiveness, as well as lessen the time and cost of clinical trials. The Principal Investigator of this initiative is Michael W. Weiner, MD, VA Medical Center and University of California – San Francisco. ADNI is the result of efforts of many co-investigators from a broad range of academic institutions and private corporations, and subjects have been recruited from over 50 sites across the U.S. and Canada. The initial goal of ADNI was to recruit 800 adults, ages 55 to 90, to participate in the research, approximately 200 cognitively normal older individuals to be followed for 3 years, 400 people with MCI to be followed for 3 years and 200 people with early AD to be followed for 2 years. For up-to-date information, see http://www.adni-info.org.

### Participants

The data used in this study were obtained from ADNI2 and ADNIGO. We identified 227 subjects with rs-fMRI data aged from 60 to 90 years. Then 59 subjects were excluded due to an unmatched time window, according to our criterion that an individual’s imaging data and clinical assessments were required to be collected within six months. Finally, the remaining 168 subjects (HC = 62, aMCI = 81, AD = 25) were used in the current study (see Table 1). Montreal Cognitive Assessment (MoCA), ADNI composite score for memory, and executive function (ADNI-Mem and ADNI-EF) were extracted from the data set to be neuropsychological assessments [34, 35].

### Imaging data acquisition

All imaging data were collected on a 3.0 Tesla Phillips MRI. The structure images were obtained by using a MPRAGE scan (TR/TE = 6.77/3.13 ms, TI = 0 ms, FA = 9°, matrix = 256×256, resolution 1×1×1mm3, slice thickness = 1 mm). The rs-fMRI imaging data were obtained by using an echo-planar imaging (EPI) sequence (TR = 3000 ms, TE = 30 ms, flip angle = 80º, slice thickness=3.3 mm, matrix=64×64, spatial resolution=3×3×3 mm^3^, number of volumes = 140, number of slices = 48).

### Brain atrophy

Voxel-based morphometry (VBM) analysis was performed using SPM8. Briefly, the structural images were segmented into gray matter (GM), white matter, and cerebrospinal fluid. After an initial affine registration of GM map into the MNI space, the GM images were generated through an iteratively nonlinear registration using DARTEL, a toolbox with a fast diffeomorphic registration algorithm [36]. For each participant, the GM map was resliced (3×3×3 mm) and smoothed (FWHM = 6 mm). Then individual’s GM map was used as a covariate for controlling brain atrophy in the following analyses.

### rs-fMRI data preprocessing

All functional images were preprocessed using DPARSF [37] based on SPM8 (http://www.fil.ion.ucl.ac.uk/spm/). The first 10 volumes were excluded to avoid potential noise related to the equilibrium of the scanner and participant’s adaptation to the scanner. For each participant, the remaining 130 volumes were corrected for slice timing and head-motion, co-registered to their structure images, and normalized to the Montreal Neurological Institute (MNI) standard space (resliced to 3×3×3 mm). Then all the imaging data were removed linear trend and filtered with a bandpass filter of 0.01-0.08 Hz. Prior to MSE analysis, nuisance covariates were regressed out, including 6 head motion parameters, white matter signal, and cerebrospinal fluid signal. All the participants exhibited head motion less than 2.5 mm or 2.5 degrees in the current study. Furthermore, given the commonality of head motion in old age and its confounding effect on resting-state functional connectivity [38], we still controlled for head motion in the following analyses.

### Multiscale entropy of BOLD signals

Since no rigorous guideline exists for choosing the parameters to calculate MSE, we first determined the proper combination of parameters by examining the MSE differences among HC, aMCI and AD groups in 90 cerebral regions based on automated anatomical labeling (AAL) template [39]. For each participant, BOLD time series of each AAL brain region was obtained by averaging the BOLD time series of all voxels within a given region, and was normalized to zero mean and unit variance. The procedure of MSE calculation has been well described in previous studies and can be summarized in three steps: (a) constructing coarse-grained time series according to different time scales; (b) calculating the sample entropy for each coarse-grained time series with suitable *m* and *r*; (c) examining the sample entropy profile in MSE analysis [40]. To capture the dynamic changes of BOLD signals at different timescales, we generated multiple coarse-grained time series by down-sampling the original BOLD time series {x_1_,…, x_i_,…, x_N_}. For time scales (τ), the coarse-grained time series {y^(τ)^} is constructed by averaging data points within non-overlapping windows as follows:

$$\;\;y_{j}^{\left(\tau \right)}=\frac{1}{\tau }\sum_{i=\left(j-1\right)\tau +1}^{j\tau }x_{j},\;\;1\leq j\leq \frac{N}{\tau }$$

With τ = 1, the time series {y^(1)^} is the original BOLD time series which represents a short time scale, whereas larger τ represents longer time scales. Then sample entropy was computed to quantify the irregularity of a time series signal with length *N* [41]. To calculate MSE, we calculated the sample entropy for each coarse-grained time series {y^(τ)^}:

$$S_{E}\left(m,\;r,\;N\right)=ln\frac{\sum_{i=1}^{N-m}\;{n^{\prime }}_{i}m}{\sum_{i=1}^{N-m}\;{n^{\prime }}_{i}m+1},$$

Where *m* is the pattern length, *r* is the similarity criterion, and ${n^{\prime }}_{i}$ is the number of matches. Sample entropy is defined as the negative natural logarithm for the conditional properties that two sequences similar within a tolerance *r* for *m* points remain similar at the next point [42].

Different parameters were applied to calculate MSE based on different modalities of data, such as *m* = 1 or 2, *r* = 0.1-0.5 in EEG data [27, 43], and *m* = 1, *r* = 0.35 in fMRI data [13, 19]. Given the short length of data in our study, we applied *m* = 1 to obtain reliable sample entropy estimation following the recommendation in previous studies [19, 44]. Using different parameters (*r* = 0.1-0.5, scale = 1-4), one-way ANOVA was applied to examine MSE differences across the HC, aMCI, and AD groups for each AAL region (p < 0.05, uncorrected for multiple comparisons to ensure the inclusiveness of relevant regions). The F scores of all the significant regions were summed to define the optimal parameters, showing larger F scores indicated better discrimination power across the three groups (see Figure 1). Then an individual’s entropy map was generated using the optimal parameters, and was smoothed (FWHM = 6 mm) to proceed with the subsequent whole-brain voxel-wise analysis. Also, each BOLD time series was normalized before generating the MSE map. A one-way ANCOVA was applied to examine the significantly different regional MSE among the HC, aMCI, and AD group using statistical toolbox in REST (http://restfmri.net/forum/index.php?q=rest), controlled for age, education, GM, and head motion. The resulting statistic map was corrected for multiple comparisons with a threshold of individual p < 0.01 with cluster size > 49 voxels (corresponding to corrected p< 0.05), determined by Monte Carlo simulations (Ledberg, Akerman, & Roland, 1998) using the AFNI AlphaSim program (https://afni.nimh.nih.gov/pub/dist/doc/manual/AlphaSim.pdf). In addition, to further examine the different MSE patterns between the AD and HC group in the temporal domain, a set of scale values (scale = 1-4) were applied to generate the entropy maps in each time scale.

### Statistical analysis

All the statistical analyses were performed using SPSS 22.0. In ANCOVA result, the peak value within each significant region was extracted for a post-hoc analysis, examining the MSE differences between each pair of groups (p < 0.05, FDR correction). Partial correlation was applied to examine the relationships between MSE and neuropsychological assessments in the entire sample (p < 0.05, FDR correction), controlled for age, education, head motion, and brain atrophy.
